# Developments on the Smart Hydrogel-Based Drug Delivery System for Oral Tumor Therapy

**DOI:** 10.3390/gels8110741

**Published:** 2022-11-15

**Authors:** Yiwen Zhao, Bei Ran, Xi Xie, Wanrong Gu, Xiuwen Ye, Jinfeng Liao

**Affiliations:** 1 State Key Laboratory of Oral Diseases, National Clinical Research Centre for Oral Diseases, West China Hospital of Stomatology, Sichuan University, Chengdu 610041, China; 2 Institute of Regulatory Science for Medical Devices, Sichuan University, Chengdu 610064, China

**Keywords:** oral tumor, stimuli-responsive, smart hydrogels, localized chemotherapy, drug delivery

## Abstract

At present, an oral tumor is usually treated by surgery combined with preoperative or postoperative radiotherapies and chemotherapies. However, traditional chemotherapies frequently result in substantial toxic side effects, including bone marrow suppression, malfunction of the liver and kidneys, and neurotoxicity. As a new local drug delivery system, the smart drug delivery system based on hydrogel can control drug release in time and space, and effectively alleviate or avoid these problems. Environmentally responsive hydrogels for smart drug delivery could be triggered by temperature, photoelectricity, enzyme, and pH. An overview of the most recent research on smart hydrogels and their controlled-release drug delivery systems for the treatment of oral cancer is given in this review. It is anticipated that the local drug release method and environment-responsive benefits of smart hydrogels will offer a novel technique for the low-toxicity and highly effective treatment of oral malignancy.

## 1. Introduction

Squamous cell carcinoma of the oral mucosa, which can affect the tongue, gums, and mouth floor, is referred to as “oral cancer” in general [1,2]. According to World Health Organization data, there were 377,713 new instances of lips and oral cavity cancer in 2020, placing them 16th among recognized malignant tumors. Unfortunately, 177,757 new deaths were reported [3,4]. The growth and invasion rates of oral tumors are relatively slow. The diseased and eroded tissues around it can be mostly removed by surgery. Therefore, clinical therapy for oral tumors at present is mainly surgical resection. The tumor tissue cannot be seen clearly during surgery due to insufficient excision and a constrained viewing field. On the other hand, an advanced oral tumor has a large volume and its cells have migrated from adjacent lymph nodes all over the body. To completely eradicate the oral tumor, advanced oral tumor treatment must be combined with postoperative radiotherapy and chemotherapy [5,6,7]. In the process of chemotherapy for oral tumors, the chemotherapeutic drugs mainly include cetuximab [8], fluorouracil [9], paclitaxel [10], docetaxel [11], cisplatin [12], and methotrexate [13]. When anticancer medications were taken orally, they caused oral cancer lesions that were exposed to cytotoxic chemicals for a long period [14,15]. However, it is believed that the medications’ poor solubility, low apparent permeability, and restricted bioavailability pose the most challenges to oral administration [16]. Intravenous administration of chemotherapeutic drugs is also the main mode of administration, which overcomes the problems caused by the diversity of intestinal mucosal absorption and can immediately obtain high bioavailability [17]. However, chemotherapy medications do not only target tumors; they also administer significant doses of medication to healthy tissues, resulting in significant harm to healthy tissue and unfavorable consequences on patients [18]. This is essential for increasing the effective residence time and medication concentration in the tumor site to enhance the therapeutic efficiency of chemotherapeutic agents and decreasing adverse side effects. A research focus and challenge can make it possible for medications to reach the lesion effectively and increase the effectiveness of its therapy.

A potential method to lessen the adverse effects on healthy tissues and enhance tumor killing is the local controlled-release medication delivery system. The local delivery system based on hydrogel is a good candidate due to its ease of use, local treatment and sustained drug release [19,20,21,22]. The hydrogel itself is biocompatible and biodegradable with moist surface and tissue affinity [23,24]. Taking the degradation process of polyester hydrogels as an example, due to their highly hydrolyzed ester linkages in the polymer backbone and their adaptation to locations of enzyme activity, polyester hydrogels are capable of undergoing hydrolysis and enzymatic breakdown. The body will not be harmed by its degradation products, carbon dioxide and water, which are eventually discharged via the body’s metabolism [25,26,27]. Drugs carried in hydrogel can be administered into the tumor region, and meanwhile, the hydrogel carrier allows a combination of multiple drugs to achieve synergistic anticancer effects with high drug loading and low dosage [28,29]. The hydrogel can be designed and prepared with smart behaviors according to different reactions to the external environment [30]. The effects of temperature, photoelectricity, and pH may be observed in smart hydrogels, resulting in changes in hydrogel properties for controllable and sustainable drug release. Thereby, this smart hydrogel platform achieves the dual controllability of space and time of the drug delivery process, improving the oral tumor treatment efficiency and reducing the side effects [19,31,32,33,34].

As a result, smart hydrogel-based drug delivery systems not only have an advantage over conventional hydrogel drug delivery systems, but they may also be utilized to treat oral tumors using various biological therapy methods by manipulating stimulation parameters. The concepts, methods of synthesis, and benefits of several kinds of smart hydrogels and their sustained-release drug delivery systems for the treatment of oral tumors are described in this review. The treatment of oral tumors and other local malignancies has demonstrated promising application potential for intelligent hydrogel-based drug delivery devices. In the ongoing study on oral tumors, certain challenges addressed by the smart hydrogel carrier were mentioned. Additionally, this study discusses the potential for clinical translation as well as the challenges that must be overcome.

## 2. The Common Types of Smart Hydrogels

**Figure 1:**
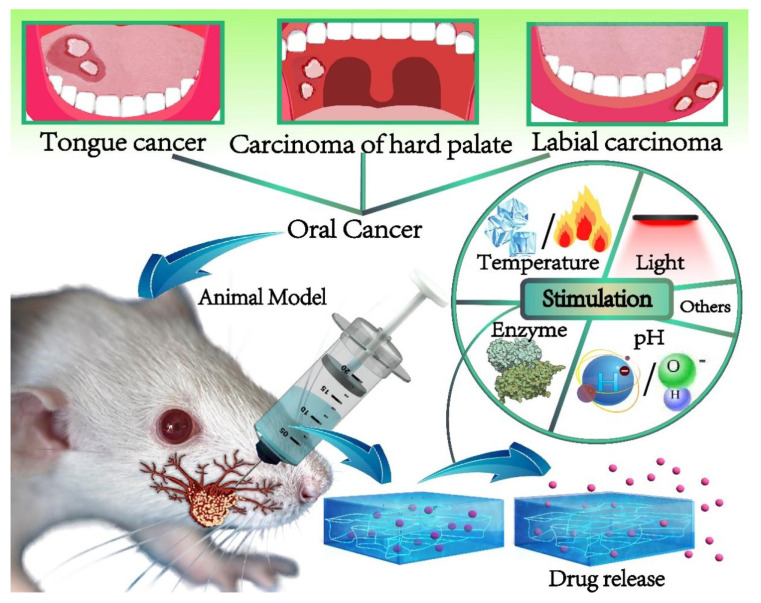
The smart hydrogel-based drug delivery system for oral cancer therapy.

In the treatment of oral tumors, smart hydrogel-based drug delivery systems can be controlled by adjusting stimulus factors. By placing hydrogels in the affected area of oral cancer, according to the different sensitivity of different smart hydrogels to different stimuli, corresponding stimuli are applied to control the local targeted transmission of drugs and other bioactive molecules, as shown in Figure 1. The four most common environmentally sensitive hydrogels are introduced as follows: thermosensitive hydrogels, photosensitive hydrogels, enzyme-responsive hydrogels and pH-sensitive hydrogels.

### 2.1. Thermosensitive Hydrogels

Thermosensitive hydrogels are one of the stimuli-sensitive hydrogels that have been the subject of most investigations. The structure of thermosensitive hydrogels can change depending on how much they expand, because the hydrophobic groups might undergo a phase transition at a particular temperature [35]. The temperature of the solution is its Lowest Critical Solution Temperature (LCST) or Highest Critical Solution Temperature (HCST) [36,37]. The most popular type of thermosensitive hydrogels is LCST. The hydrophobic contact between polymer chains weakens when the surrounding temperature is lower than LCST. This makes it simple to dissolve the therapeutic drug in the solution and inject it directly into the tumor site while it is still in solution form. The hydrophobicity of hydrogel polymer chains increases noticeably at temperatures greater than LCST. According to Prausnitz’s theory [38], hydrogen bonds between hydrophilic segments and water molecules in polymer chains predominate at low temperatures. Stretching molecular chains causes polymers to create a network structure, and water molecules scatter in the network structure in a swelling state. However, when the temperature rises, the photophilic effect between the polymer chain segments increases while the hydrogen bond effect diminishes. The network structure contracts, reflecting the overall impact, which causes water molecules to extrude, medications to be released from their capsules, and the gel to constrict. The hydrogel drainage shrinkage makes the structure compact, and the gel state can be formed at physiological temperature in situ [39,40,41,42]. For oral tumor treatment, the hydrogel can sustainably release the medications that have been encapsulated.

The ability to be employed in intratumoral and peri-tumor injection, which may effectively increase the number of medications in the tumor tissue and prevent systemic toxicity, is one of the benefits of injectable thermosensitive hydrogels [36,43]. After the injection of thermosensitive hydrogels into the oral cavity, the hydrogels change from a solution state to a gel state at body temperature. The hydrogels can stay in the periodontal pocket for a long time and release the drug continuously at the same time for the treatment of oral tumors [44,45]. For instance, protein-based chemotherapeutic drugs are easily degraded by enzymes. When they enter the body, their efficacy is not fully exerted. Furthermore, due to surface activity, selective exclusion, steric restriction of protein–protein interactions, or through increasing viscosity and reducing protein structural mobility, hydrogels can stabilize proteins and peptides [46,47]. With the help of temperature-sensitive hydrogel delivery systems, the bioavailability of the proteins can be significantly increased, thus making up for the limitations of traditional oral cancer treatment.

The macromolecules that make up thermosensitive hydrogels might be man-made or biological. Natural macromolecules include hyaluronic acid, chitosan, gelatin, and others. The synthetic polymers Poly(N-isopropyl acrylamide) (PNIPAAm), Poly(ε-caprolactone)-poly(ethylene glycol)-poly(ε-caprolactone) (PCL-PEG-PCL), Poly(caprolactone)-b-poly(ethylene)-b-poly(caprolactone) (PEG-PCL-PEG, PECE), poly(lactide) -b- poly(ethylene glycol)-b- poly(lactide) (PLA-PEG-PLA), and other block copolymers are employed in the production of smart hydrogens. A type of temperature-sensitive polymer made from N-isopropyl acrylamide is one of them, and it is called PNIPAAm. PNIPAAm-based thermoresponsive hydrogels are the most representative thermosensitive hydrogels, which are promising drug delivery materials [48,49,50]. At lower temperatures, the drug can be loaded directly into the PNIPAAm hydrogel, injected into the periodontal pocket and gelatinized. In addition, temperature-related drug release is provided in a slow and linear manner [51,52]. PNIPAAm can be combined with other polymers to adjust LCST to meet different phase transition requirements [53,54,55]. For instance, the hydrogels created by mixing HA and PNIPAM improve the microstructure and mechanical–chemical property interactions, which reduce dehydration and unnecessary syneresis of PNIPAAM and improve cell adhesion. In addition, the mechanical properties of the hydrogel were improved by adding chitosan-g-acrylic acid-coated PLGA (ACH-PLGA) particles and using the allyl group of the chitosan-g-acrylic acid shell as the PNIPAM’s crosslinking agent [56]. Thus, PNIPAAM can be well adapted to the complex microenvironment of oral cancer by binding to other polymers. After the preparation of thermosensitive hydrogel, dynamic light scattering (DSC) may be used to explain how micelle size and size distribution change with temperature [57], and the rheologies of thermosensitive hydrogels can be analyzed by the tube-inverting method to determine the critical gelation temperatures [58]. The transition temperature and gel enthalpy can be determined using the endothermic peak produced by differential scanning calorimetry. Additionally, the use of ultrasonic velocity, dynamic and static light scattering, small-angle neutron scattering (SANS), rheology, dielectric constant measurement, and microcalorimetry may be used to examine the phase transition and mechanism of the solution and gel states [59]. Xia et al. mixed PNIPAAm with alginate to produce lignin-based thermosensitive hydrogels with high mechanical strength [60]. The hydrogel loaded with doxorubicin (DOX) achieved sustained release and had obvious cytotoxicity to tumor cells [61]. Alginate-g-PNIPAAm was synthesized by chemical interaction by Liu et al. [62]. Diblock copolymers formed by hydrophilic alginate and hydrophobic PNIPAAm can form micelles, and then the micelles are aggregated to form hydrogels. The copolymer was mixed with DOX solution at low temperatures and self-assembled micelles to encapsulate DOX. The continuous release of DOX from micelles and the formation of hydrogels at body temperature showed the potential to avoid systemic adverse effects in the treatment of oral tumors.

### 2.2. Photosensitive Hydrogels

One type of intelligent hydrogel is photosensitive hydrogel, which can alter the chemical and physical characteristics of hydrogels using various light signals. When photosensitive radicals are ionized in the presence of light, the osmotic equilibrium of the gel medium is upset, which causes ions and water to move into or out of the gel network due to an imbalance in permeability. As a result, the gel either swelled or contracted [63,64,65]. The skeleton and photosensitive portion make up the most photosensitive hydrogels. The photosensitive portion is in charge of detecting light signals and translating them into chemical signals. After the light reaction including isomerization, pyrolysis, or dimerization, the structure and property of hydrogels were changed [66,67,68].

**Figure 2:**
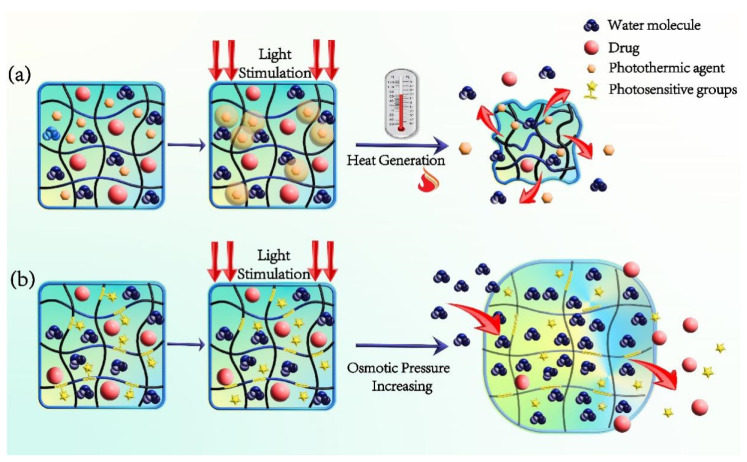
Drug release mechanism of the photosensitive hydrogel. (**a**) The photosensitive hydrogel containing photothermal agent; (**b**) Introducing photosensitive groups onto the chain of the gel polymer.

Photosensitive hydrogels can be obtained by the following methods (Figure 2). One is to add photosensitive groups to the gel polymer’s main chain or side chains, allowing them to become photosensitive so they can polymerize into hydrogels [69,70]. The photosensitive groups dissociate when exposed to visible or ultraviolet light, because the chemical bonds between the photosensitive groups and the hydrogel’s polymer chains are broken. This increases the osmotic pressure inside the hydrogel, allowing external water molecules to enter, expanding its pores and making it easier for drugs to escape through the pores [71,72]. The other is to introduce photosensitive nanomaterials (like gold nanorods, graphene, and CuS nanoparticles) into the gel at the same time. The photothermal agents can absorb the light energy and make the hydrogel warm up locally. As the temperature rises, the hydrogels’ protophilic effect between their polymer chain segments of the temperature-sensitive group grows while the hydrogen bond effect weakens. Due to the contraction of the network structure, water molecules and medications are extruded from their capsules [73]. After the preparation of photosensitive hydrogel, for rheological investigation, it is possible to compare the viscosity distribution at various temperatures using a cone plate rheometer [74]. Transmission electron microscopy (TEM), dynamic light scattering (DLS), and scanning electron microscope (SEM) may all be used to examine the hydrogel’s microstructure and size. UV-VIS-NIR spectrophotometers can record the near-infrared photosensitive hydrogel’s UV-VIS-NIR absorption spectra [66]. Under the assumption of laser irradiation, the process of temperature rise may be seen using an infrared thermal imager in order to identify the photothermal conversion impact of hydrogel [75].

Photosensitive hydrogel provides a new possibility for drug delivery in oral cancer chemotherapy due to its noncontact degradation with high spatial and temporal resolution [76]. The photosensitive hydrogel can be used to release the drug by the photothermal effect of gel–sol phase transformation, and self-assembly photosensitive hydrogel can be used to release the drug through photopolymerization or photolysis [77]. Therefore, photosensitive hydrogel is considered an ideal carrier for smart drug delivery. For instance, photothermal agents sensitive to near-infrared light can trigger the photosensitive phase transition of hydrogel gel–sol by converting light into heat, so that the hydrogel network can be partially destroyed by near-infrared light at different times, so as to accurately control drug release [78,79,80]. Or, the nanostructure of drug-loaded hydrogel can be softened or melted by the rise of temperature, which can also achieve the goal of controlled drug release [81]. Oral cancer cells are less resistant to heat than healthy tissue. Therefore, the combination of photothermal therapy and chemotherapy by photosensitive hydrogel is a good way to kill the oral tumor efficiently.

### 2.3. Enzyme-Responsive Hydrogels

Enzyme-responsive hydrogels, as a typical kind of environment-responsive hydrogels, are formed or degraded by the catalysis of related enzymes [82,83]. Enzymes are highly selective for the substrate of the catalytic reaction. Compared with other chemical conventional reactions, the action conditions of enzymes are mild. They are suitable for the temperature of about 37 °C in the body as well as a neutral, weak acid, weak alkaline humoral environment [84,85,86]. These advantages enable enzyme content and activity to be used as primitive biological triggers to realize the locally controlled release of enzyme-mediated drugs. Commonly available enzymes used as reaction triggers enzyme-responsive hydrogels include matrix metalloproteinase enzymes (MMP) [87], phosphatase [88], trypsin [89], and tyrosinase [90]. MMPs, which are a group of endopeptidases that can cleave peptide bonds [91,92], are highly expressed in the oral cancer microenvironment compared to normal tissues [93,94], and play key roles in angiogenesis, invasion, and metastasis [87,95,96]. In the synthesis of hydrogels, MMP-sensitive peptide is used as the crosslinking agent. It can form a biodegradable system under the influence of MMP. In order to create MMPs-sensitive hydrogels, certain peptide-bound amino acid fragments that are reactive with MMPs are typically crosslinked with polymer chains. Enzymatic cleavage of these crosslinkers causes drug release and polymer network decomposition as a result of matrix degradation [97].

In order to successfully design and prepare functional enzyme-responsive hydrogels, three general requirements must be met. To begin with, the hydrogel system must have recognition components or substrate imitators that enzymes can uniquely recognize. Second, the enzyme must come into touch with the attached substrate, which has a significant impact on the kinetics of the enzyme-catalyzed process. Thirdly, the hydrogels’ characteristics ought to alter as a result of the enzyme substrate reaction. Chemotherapeutic chemicals can be physically encapsulated in hydrogels or can interact with them covalently to distribute them locally through changes in hydrogel shape or breakdown caused by enzymatic activity [86,98,99]. After preparing the enzyme-sensitive hydrogel, in addition to measuring the porosity, size, and other conventional characteristics, it is also necessary to test its sensitivity to related enzymes [100]. The degradation of gel can be stopped by mixing the relevant enzyme with the hydrogel, freezing the gel within a predetermined time interval, and then running gel permeation chromatography to check the molecular weight distribution of the degraded polymer [83].

### 2.4. pH-Sensitive Hydrogels

pH-sensitive hydrogels are polymer gels, which can vary with the pH and ionic strength of the environment [101,102,103]. The tumor microenvironment becomes acidic when tumor tissue undergoes anaerobic glycolysis, which lowers the pH of the tumor tissue relative to healthy tissue [104]. The tumor’s external environment has a pH of around 6.5, whereas its intracellular environment has a pH of about 5.0 [105,106]. The consumption of glucose and the hydrolysis of glutamine are considerably increased in the tumor microenvironment of oral squamous cell carcinoma (OSCC), which increases the generation of lactic acid and decreases the local pH [107]. As a result, the pH differential between the normal tissue and the oral tumor microenvironment can influence the release of chemotherapeutic medicines in pH-responsive hydrogels.

Anionic-cationic pH-responsive hydrogels and covalent pH-responsive hydrogels are the two main categories of pH-responsive hydrogels. When the pH of the surrounding environment rises, the negatively charged acidic components of polyanions in anionic hydrogels start to resist one another. At the same time, hydrogen bonds are created between ionized groups and water molecules, weakening the hydrophobic contact between chains. Additionally, the hydrogel structure’s osmotic pressure rises due to the creation of additional ions as a result of dissociation. Water enters the structure due to the hydrogel’s permeability gradient between its inner and outer layers, which causes the matrix to enlarge [108,109,110]. When pH is lower than pKa, the anion groups are protonated, which increases the hydrophobicity of polymer chains and makes the hydrogel system shrink [111,112]. On the contrary, the swelling of cationic hydrogel increases when the pH value is lower than pKa. The shrinkage of cationic hydrogel occurs when pH is higher than pKa [113]. In order to achieve a precise medication release, the hydrogel’s swelling behavior may thus be modified by the pH of the tumor microenvironment [114]. Covalent bonds that are sensitive to pH, such as ketals and hydra-zone bonds, are present in another pH-responsive hydrogel [115,116,117]. Due to pH variations, these covalent connections can be disrupted, and the hydrogel’s structure can deteriorate. This structural feature has made pH-responsive hydrogels popular in the study of intelligent drug delivery systems under certain pH settings. These hydrogels are less prone to damage from tissue movement and have more stable mechanical characteristics than the anionic and cationic pH-responsive hydrogels. Taking into account the obvious movement of oral tissue during daily activities, the drug delivery system of covalent pH-responsive hydrogel in oral cancer chemotherapy should be more stable [118].

## 3. The Application of Smart Hydrogel-Based Drug Delivery Systems in Oral Tumor Therapy

Hydrogel systems are efficient functional media for drug delivery because of their minimal invasiveness, simplicity in formulation, and potential for regulated long-term drug release [119,120,121]. Additionally, hydrogel-based therapeutic systems have demonstrated strict restrictions on the distribution of target tissues, allowing targeted therapy. This is crucial for anticancer medications that are linked to serious side effects when given systemically and are therefore particularly important. In view of the toxicity of chemical drugs to normal tissues and organs, the hydrogel local delivery system has become an important means of oral cancer treatment. Intelligent hydrogels used in the hydrogel delivery system can respond to environmental stimuli (like heat, pH, light, and ultrasound) to achieve in situ gelation and controlled release, greatly improving the convenience and effectiveness of drug delivery. This enables the controllability of the hydrogel delivery system and the customized treatment of oral cancer.

### 3.1. Application of Drug Release System Based on Thermosensitive Hydrogels

Drug transport and hydrogel degradation are the fundamental tenets of thermosensitive hydrogel administration [122,123]. For instance, poly(D, L-lactide-coglycolide)-poly(ethyleneglycol)-poly(D, L-lactide-coglycolide) triblock copolymers (PLGA-PEG-PLGA) are injectable thermosensitive hydrogels that undergo a sol–gel transition at body temperature [124,125]. Nevertheless, the wide pores and high water content of injectable hydrogels may be to blame for the first abrupt release of loaded medicines.

**Figure 3:**
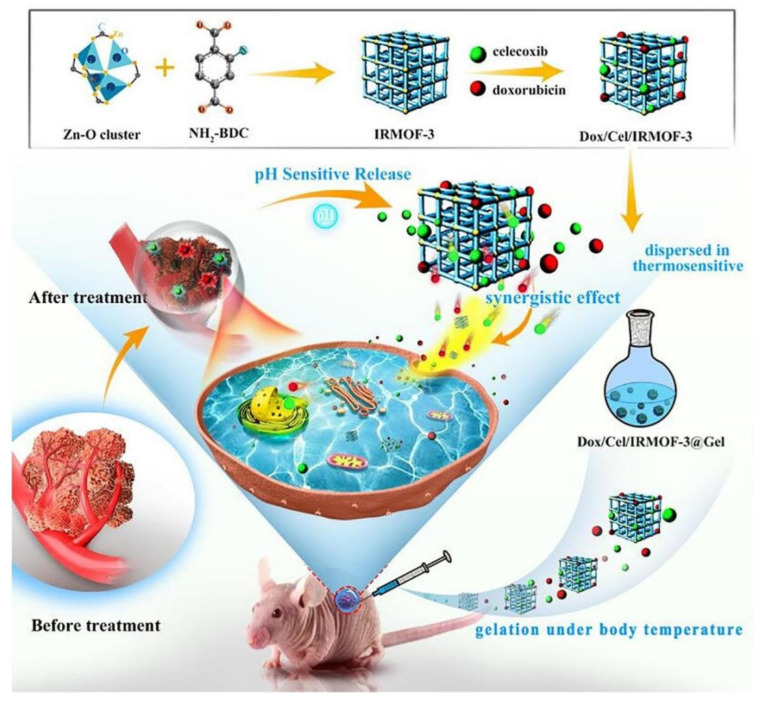
Schematic illustration of Dox/Cel/MOFs@Gel as novel injectable metal–organic frameworks@thermosensitive hydrogel local dual drug delivery for oral cancer therapy. Reprinted with permission [109].

An injectable thermosensitive hydrogel consisting of an organic skeleton (MOF), DOX and celecoxib (designated as Dox/Cel/MOFs@Gel) can solve the above burst release problems for the treatment of oral cancer (Figure 3) [126]. In this hydrogel system, integrating MOF into thermosensitive hydrogel can slow down the release rate of Cel and significantly reduce the sudden release effect. IRMOF-3 can be used as a protective layer of DOX to prevent its rapid high dissipation. Thus, DOX achieved prolonged release from the Mofs@gel in comparison with Gel alone. Additionally, the anti-squamous cell carcinoma-9 effect of this hybrid hydrogel system was demonstrated with effective tumor suppression [127].

**Figure 4:**
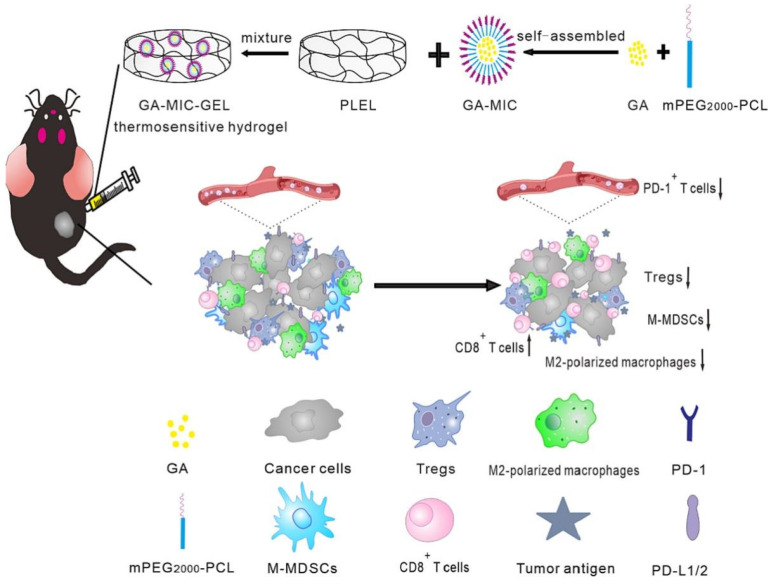
Schematic diagram of the in situ thermosensitive hydrogel containing GA micelles for improving anti-tumor immunity against OSCC. The GA micelle-encapsulated PLEL sol was locally injected into the tumor, formed hydrogel at body temperature, and continually released GA in situ, thus exerting the chemotherapeutic effect and anti-tumor immune activation. Reprinted with permission [128].

Furthermore, Chen et al. constructed gambogic acid (GA)-loaded thermosensitive hydrogel and applied it locally to the treatment of OSCC (Figure 4) [128]. GA-loaded mPEG 2000-PCL micelles (GA-MIC) and Poly(D,L-lactide)-b-Poly(ethylene glycol)-b-Poly(D,L-lactide)(PDLLA-PEG-PDLLA, PLEL) were physically mixed to form injectable thermosensitive hydrogels. Among them, GA-MIC can improve the water dispersion and stability of GA, and GA-MIC-GEL can form hydrogels in situ. The experiment demonstrated a strong anti-tumor impact of local GA administration on both primary and metastatic cancers. Additionally, the animals given GA-MIC-GEL demonstrated a rise in cytotoxic T cells and a decrease in immunosuppressive cells at the tumor site, demonstrating the reversal of T-cell activation and the immunological milieu around the tumor. It can be concluded that local GA-MIC-GEL treatment can enhance the systemic anti-tumor response by altering the tumor immune microenvironment. This study shows a new anti-tumor mechanism of GA and provides a way to develop a local delivery method for the treatment of systemic cancer.

Li et al. created an intratumoral thermosensitive hydrogel made of poly(ethylene glycols), poly(Ɛ-caprolactone)-poly(ethylene glycols), and simultaneous loading of suberoylanilide hydroxamic acid (SAHA) and cis-platin (CDDP) in consideration of the synergistic anticancer and drug-resistance reduction effect of hydrogels loaded with multiple drugs [129]. Since they contained both the hydrophobic PCL block and the hydrophilic PEG block, PECE triblock copolymers had an amphiphilic nature. In PECE copolymers, sol–gel–sol phase transitions with temperature dependency were observed. The PECE copolymer’s aqueous solution flowed well at low temperatures, but at about 37 °C, it developed a gel phase [130]. When PECE hydrogel was injected into OSCC mouse tumors, the loaded SAHA and CDDP could be released for more than 14 days, improving the therapeutic impact and minimizing adverse effects [131].

### 3.2. Application of Drug Release System Based on Photosensitive Hydrogels

Photostimulation is a fast, non-invasive, safe and easy-to-adjust strategy in the stimulation mode of environment-responsive hydrogels. Drug release from hydrogels can be controlled by simply turning on or off lights and adjusting irradiation parameters without the need for additional reagents. This makes photosensitive hydrogels a promising approach for the targeted drug delivery of oral cancer.

Chen et al. synthesized a newfangled ink@hydrogel consisting of FeCl_3_, traditional Chinese ink (Hu Kaiwen ink), and agarose hydrogel to carry dihydroartemisinin (DHA) in cancer therapy [132]. DHA has been studied as an alternative tumor therapeutic agent. Through a high concentration of ferrous iron, the homolysis of a weak endoperoxide bridge can be accelerated to produce active oxygen free radicals, and various tumor cells can be killed in vitro and in vivo [133]. Ink in ink@hydrogel converts light into heat when exposed to radiation at 1064 nm for a short period of time, and hyperthermia causes the reversible hydrolysis of hydrogels. Fe^3+^ ions migrate from the hydrogel to the tumor microenvironment and are changed to Fe^2+^ to promote the dissolution of the endoperoxide bridge in the pre-injected DHA. Free radicals are released as a result, and they have an effective anticancer effect.

Considering that light itself, as the stimulation source of photosensitive hydrogels, has a killing effect on head and neck squamous cell carcinoma (HNSCC) [134,135,136,137], researchers usually use chemotherapy in combination with photodynamic therapy (PDT). Combining chemotherapy drugs can overcome the limitation of poor light penetration in PDT and increase the sensitivity of cancer cells to hyperthermia and reactive oxygen species (ROS), while PDT, because of its broad-spectrum activity, is essential for killing cancer cells that are resistant to other forms of treatment [138]. Intracellular trafficking of co-delivered chemotherapy drugs delivered via nanoparticles can be enhanced by PDT and drug efflux p-glycoprotein pump activity in drug-resistant cells can also be inhibited [139,140], to further improve the efficacy of delivered chemotherapy drugs.

Sun et al. prepared a dynamic covalent hydrogel with the ability of near-infrared light triggering drug release (GelPV-DOX-DBNP) [141]. Hybrid gels made of fructosyl sugar polymer (PolyFru) and BOB-modified hyaluronic acid (BOB-HA) are made via particular carbohydrate interactions with benzoxyheterocyclopentane in aqueous solutions. After that, the gel is combined with the photosensitizer perylene diimide zwitterionic polymer (PDS) [142], and reductant Vitamin C is added to the gel to create near-infrared light-induced degradable hydrogel (GelPV). This hydrogel can spontaneously cause the formation of hydrogen peroxide to break the dynamic covalent bond based on benzoxyheterocyclopentadiene carbohydrate interaction, resulting in the release of DOX and photothermal. After the released DB-NPs enter the interior of the tumor, it generates heat under NIR irradiation to kill local tumors.

**Figure 5:**
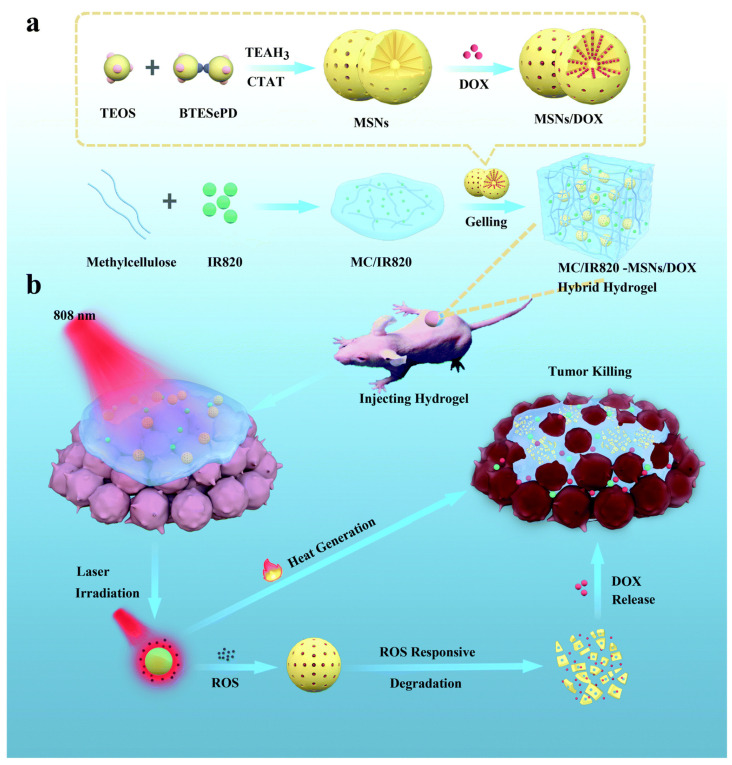
(**a**) The preparation of NIR-responsive MC/IR820-MSNs/DOX and (**b**) its use in the localized synergistic photochemotherapy of OSCC. Reprinted with permission [143].

For synergistic OSCC photochemotherapy, Wu et al. created a diselenide-bridged-MSN-containing hybrid hydrogel platform (Figure 5) [143]. Methyl cellulose (MC) hydrogel is mixed with mesoporous silica nanoparticles (MSNs) that have been DOX-loaded in addition to being encapsulated in the photothermic agent IR820 (a novel green cyanine dye). The addition of MSNs enhances the stability and mechanical performance of the hydrogel system and promotes the gel process. Excess ROS can break redox-sensitive diselenide links when exposed to NIR light, hastening matrix breakdown and MSN DOX release. A significant quantity of heat energy may also be released by the integrated IR820 when the laser stimulates it, which can be used to thermally destroy tumor cells. In the experiment, it is proved that the toxicity of the hydrogel system is low, and the killing effect is prolonged due to the continuous release of drugs, thereby achieving efficient synergistic photochemical therapy.

Photodynamic chemotherapy based on hydrogel has been utilized extensively in the treatment of breast cancer [144], prostate cancer [145], skin cancer [146], glioblastoma [147], etc. However, there are only a few studies on its application in oral cancer, which shows potential research prospects.

### 3.3. Application of Drug Release System Based on Enzyme-Responsive Hydrogels

The stroma and parenchyma of ameloblastoma and adenomatoid odontogenic tumors both exhibit high levels of matrix metalloproteinase MMP-1 expression, and MMP expression levels in cancer cells continuously increase as oral cancer progresses [93]. The expression of MMP-2 varies between adenomatoid odontogenic tumors and ameloblastoma, with 80% and 60%, respectively, immunological reactivity of tumor cells. The aforementioned two types of oral cancer had MMP-9 immunoreactive parenchymal and stromal cells [148]. Therefore, it is very meaningful to develop MMP-responsive hydrogel for local chemotherapy of oral cancer.

An MMP-2-sensitive injectable hyaluronic acid (HA) hydrogel was created by Wei Li et al. for local OSCC chemotherapy [149]. Before being loaded into HA hydrogel for in situ injection, doxorubicin (DOX) was first put in biodegradable poly (D, L-lactide)—poly (ethylene glycol)—poly (D, L-lactide) (PDLLA-PEG-PDLLA) micelles. Through Michael, matrix metalloproteinase-2 (MMP-2) response peptide initiated this crosslinking. As a result of the elevation of MMP-2 expression in OSCC lesions, MMP-2 can cleave the sequence (GPQGIWGQ) and two cysteine (C) residues in the matrix metalloproteinase-2 (MMP-2) response peptide. The loaded NanoDOX is released as a result of the continuing degradation of the hydrogel’s structure. Last, but not least, free NanoDOX functions as a chemotherapeutic drug by diffusing into cancer cells.

The “HyMic” hydrogel, created by Najafi et al., is an enzyme-responsive hydrogel made of micelles with core-cross-linked (CCL) flowers on them. Native chemical ligation is the main crosslinking method used to create P(NIPAM-co-HPMA-Cys)-PEG-P(NIPAM-co-HPMA-Cys) and P(NIPAM-co-HPMA-ETSA)-PEG-P(NIPAM-co-HPMA-ETSA)(PNE). The MMP response sequence is represented by the chosen peptide block (Lys-Gly-Pro-Gln-Gly-Ile-Phe-Gly-Gln-Lys). HyMic can be broken down by enzymes and converted into CCL micelles, which HeLa cells may take up. These findings imply that HyMic has a large potential for CCL micelle continuous release for intracellular drug delivery in MMP upregulated tissues, such as oral cancer tissues [150].

However, in the treatment of oral cancer, a single strategy often cannot completely eliminate the tumor, so combination therapy has shown great potential for cancer treatment. For the treatment of squamous cell carcinoma-15 (SCC-15), Wang et al. designed nano DOX-ICG MMP-responsive hydrogel (NDIMH), which can be injected into the oral cancer site in situ. Because the oral cancer site can secrete a large amount of MMP, the hydrogel can respond to the oral cancer site and accelerate its degradation of itself, thereby achieving the controlled release of drugs [151]. In each series of studies, NDIMH ^+^ laser with MMP-2 exhibited the greatest cytotoxicity against SCC-15 cells. The effectiveness of tumor inhibition can also be improved while reducing heart toxicity and skin phototoxicity side effects by combining phototherapy and chemotherapy.

### 3.4. Application of Drug Release System Based on pH-Sensitive Hydrogels

Numerous investigations have demonstrated that the oral cancer TME has an acidic microenvironment, which distinguishes it from normal tissues. Therefore, hydrogels with the appropriate pH can be created to release oral cancer medications based on polymers having ionizable chemical groups or polymer systems with acid-sensitive linkages.

**Figure 6:**
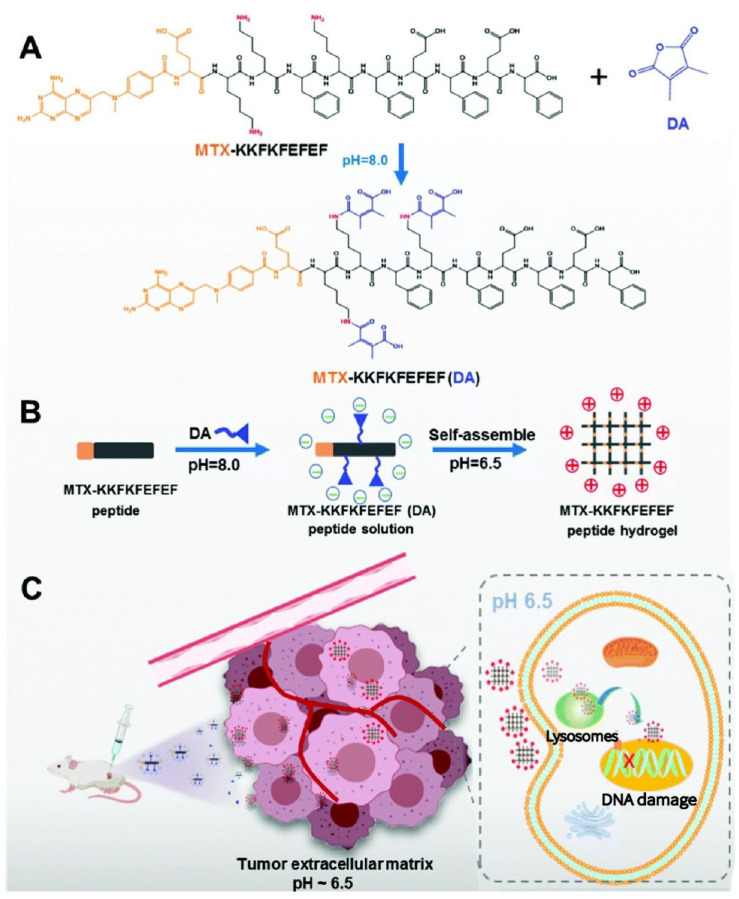
Schematic illustration of the formation of (**A**,**B**) the pH-responsive peptide hydrogel and (**C**) the anti-tumor mechanism of the pH-responsive peptide hydrogel at the tumor site. Reprinted with permission [152].

For local tumor treatment, the injectable, pH-responsive, in situ self-assembled drug-peptide hydrogel (MTX-KKFKFEFEF(DA)), as seen in Figure 6, was developed by Zhang et al. [152]. Methotrexate (MTX) and the pH-responsive linker 2,3-dimethylmaleic anhydride (DA) are joined to the peptide chain of KKFKFEFEF by an amidation mechanism [153]. The negatively charged drug peptide (pH 7.4) can be activated under the acidic microenvironment (pH 6.5), becoming positively charged and realizing the sol–gel phase transition, which results in effective cell uptake and endocytosis. This happens under the influence of charge effect and hydrogen bond, as a result of intermolecular rearrangement and assembly in the process of pH change [154].

N-carboxyethyl chitosan (CEC), produced in an aqueous solution by the Michael reaction, and dibenzaldehydeterminated poly(ethylene glycol), were combined to create a pH-responsive hydrogel by Qu et al. The hydrogel realizes the release of drugs by reversible association/dissociation of Schiff base linkages through pH stimulation, which experimentally verified the feasibility of this pH-responsive hydrogel for in vitro injection and in vivo subcutaneous injection, and the drug release experiments of doxorubicin loaded in different pH environments; the hydrogel also shows a good, sustained drug-release effect [155]. It is considered to have the prospect of being a long-acting implant for oral cancer chemotherapy-photothermal synergistic cancer treatment.

Self-healing hydrogels, as opposed to conventional injectable hydrogels, can be injected by applying shear strain/stress forces, and they immediately revert to the normal gel phase [156]. They can prevent the possible risk of drug diffusion, which efficiently uses pharmaceuticals and reduces the toxicity to normal tissues [157], as well as automatically repair damage and lengthen their service life during usage [158,159,160]. A new injectable CS/HA/GP hydrogel was created by Zhang et al. [119]. By creating reversible chemical connections between chitosan with a carboxyethyl modification (CEC) and hyaluronic acid with an aldehyde modification, hydrogels were created (A-HA). The hydrogel may swiftly self-heal without any external stimuli because of the dynamic equilibrium of the Schiff base connection between the amine group on CEC and the aldehyde group on A-HA. Its anticancer medications may release when an acidic tumor is stimulated, and diffusion release is the primary method. HA is the main ligand of the CD44 receptor. Because CD44 is highly expressed in oral cancer cells [161,162,163], the addition of HA makes the hydrogel more binding to oral cancer cells and increases the immobility of the hydrogel formed in situ.

### 3.5. Smart Composite Hydrogels for Oral Tumor Therapy

When subjected to particular environmental stimuli, stimulus-responsive polymers modify their physical characteristics. Physical characteristics including solubility, volume, polarity, and others will change when the polymer is stimulated over the critical point. These polymers shift back to their original physical characteristics when the stimulus is removed [164,165]. The most often employed triggers among them are temperature and pH since they are not only simple to adjust but frequently have a tight connection to a number of physiological activities of the organism [166]. A kind of temperature- and pH-sensitive hydrogel was prepared by Natnicha Jommanee et al. [110], who created a type of temperature- and pH-sensitive hydrogel by first creating a number of temperature-responsive diblock copolymers using poly(ethylene glycol) methyl ether (mPEG) and -caprolactone (CL), and then grafting these diblock copolymers onto pH-responsive chitosan. The hydrophilic-CL hydrophobic balance of mPEG can be used to modify its LCST [167,168]. Besides, chitosan is soluble in dilute acidic solutions with a pH lower than 6.0 due to its ionic primary amine groups with pKa of 6.3 [169]. A low pH causes amino groups to protonate and turn into -NH^3+^, which strongly repels cationic charges electrostatically and makes them water-soluble. The amino group deprotonates and changes to -NH2 when the pH exceeds the pKa. A spherical collapse structure and water repulsion are produced by the hydrophobic contact and hydrogen bonds between the polymer molecules, rendering the polymer impermeable in water. These chitosan-graft-(mPEG-block-PCL)(chitosan-g-(mPEG-b-PCL)) graft copolymers exhibit a variable temperature and pH response to the sol–gel phase transition that is in good agreement with body temperature and pH in the acidic tumor microenvironment. The method simultaneously demonstrates the persistent release of curcumin and doxorubicin for up to two weeks.

Some pH- and temperature-sensitive hydrogels can be used for the combined application of photothermal therapy (PTT) and multidrug chemotherapy. Ghavami Nejad et al. encapsulated the dopamine nanoparticles and adriamycin with a photothermal effect in acid-responsive pNIPAAm-co-pAAm hydrogel [170]. The pH-dependent medication release in a cancer environment and heat creation based on NIR laser exposure are both features of the smart composite hydrogel. Because the dopamine nanoparticles have a good photothermal conversion effect, the amide bond in the pNIPAAm-co-pAAm hydrogel and the adriamycin Brown movement increase, which promotes the release of the system in the system when the internal temperature of the gel increases. At the same time, the protonation of bortezomib under acidic conditions makes it possible to realize the combined treatment of photothermal chemotherapy through the weak acid’s sudden release in the tumor area.

**Figure 7:**
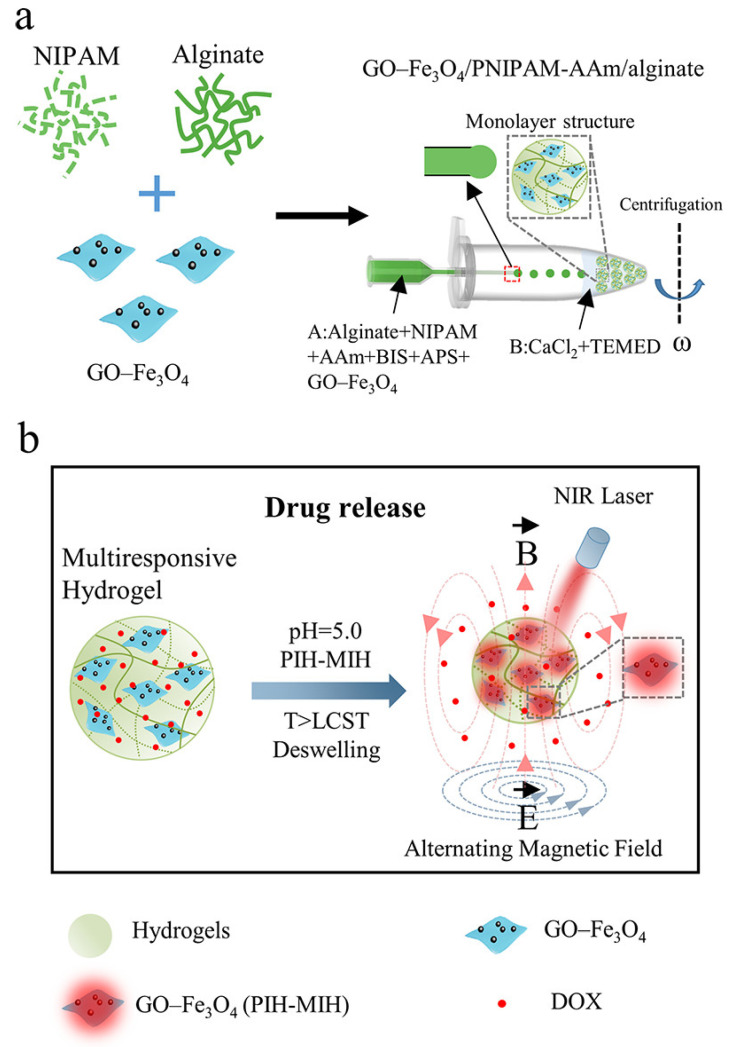
Schematic illustration of GO−Fe_3_O_4_/PNIPAM-AAm/alginate nanocomposite hydrogel microcapsules for controlled drug release. (**a**) Schematic diagram of the fabrication of GO−Fe_3_O_4_/PNIPAM-AAm/alginate NCH microcapsules based on the centrifugal microfluidic method. (**b**) GO−Fe_3_O_4_/PNIPAM-AAm/alginate NCH microcapsules for NIR light-, magneto-, and pH-responsive drug release. Reprinted with permission [176].

Although responsive hydrogels have superior performance, there are still major challenges in drug delivery efficiency when environmental stimulation is weak [171]. In order to increase the adaptability and flexibility of responsive hydrogels, researchers have suggested using nanocomposite hydrogels, which include responsive nanostructures into hydrogels [172,173,174,175]. GO-Fe_3_O_4_/poly(N-isopropylacrylamide)/alginate nanocomposite hydrogel microcapsules for controlled drug release were successfully created and fabricated by Yuan et al. (Figure 7) [176]. The hydrogel microcapsules are near-infrared light, magneto, and pH-sensitive. Under near-infrared light and an alternate magnetic field (AMF), Fe_3_O_4_ composites can efficiently convert energy into heat, driving the deflation of thermosensitive hydrogels and promoting the release of encapsulated doxorubicin (DOX) [177]. Additionally, the deswelling behavior of stimulus-responsive hydrogels may be later recovered after NIR and AMF are off; at the same time, drug release will be terminated. It is important to note that adding sodium alginate to PNIPAM hydrogel makes the drug release relatively low at pH 7.4 and quicker at pH 5.0. The considerable increase in DOX release behavior in acidic circumstances is mostly ascribed to protonation of carboxyl groups in alginate, endowing alginate with hydrophobicity, which may contribute to deformation of nanocomposite hydrogels (NCHs) microcapsules, hence promoting the DOX diffusion rate in NCH microcapsules. Furthermore, at pH 5.0, DOX release rises due to an increase in DOX solubility in an acidic environment. Finally, the multifunctional endows nanocomposite hydrogels platform combines the properties of near-infrared light, magnetic response, and pH response, offering the chance to regulate drug release in time and dose. Oral cancer therapy requirements may be met by the system’s design, which can achieve effective release under low-pH and high-temperature external physical field circumstances.

## 4. Prospective and Conclusions

The unique properties of hydrogels make them effective mediums for drug delivery. Because of the limitations of traditional chemotherapy and the toxicity of chemotherapeutic drugs to normal tissues and organs in oral cancer, the smart drug delivery system based on hydrogels has become a popular research direction. Related studies have mainly focused on the methods of sustained-release drug delivery and stimulation response [178,179,180]. The smart hydrogels have precise reactions to temperature, pH, and tiny changes in light. To ensure that the medication is delivered to the cancer tissue efficiently and at the right moment, these characteristics may be employed as trigger factors for drug release and can regulate the release curve in real time. This enables the controllability of chemotherapeutic medicines in time and space. The variations among the different kinds of stimulus-responsive hydrogels are displayed in Table 1. The loop of care can be closed at the point of care (PoC) when utilized in conjunction with pertinent point-of-care (PoC) diagnostic tools (such as biosensors sensitive to tumor biomarkers) [181]. In oral cancer treatment, this will automatically adjust the release of drugs in smart hydrogels according to specific biomarkers of real-time diseases.

The smart hydrogel-based drug delivery system must enhance certain hydrogel system attributes, such as biodegradability and biocompatibility, optimal mechanical properties, uniform drug distribution, and decreased initial release, in order to meet the demands of the human body. The thermally sensitive matrix based on PEG/polyester copolymer and POPs is the most often used smart hydrogel in compliance with these standards due to its biodegradability and extended drug release curve. A variety of drugs can have their release time controlled by the thermosensitive hydrogel system. The most prevalent stimulant is temperament. The multi-stimulus smart hydrogel, which may combine chemotherapy with other treatment modalities and have synergistic effects with anticancer medications, is the most promising therapeutic option for oral cancer. The delivery of medication at a distance can also be controlled using intelligent hydrogels that react to external stimuli. Smart hydrogels that are responsive to biological stimuli (such as enzymes or antibodies) open up the possibility of precisely targeting tumor tissues for therapy with highly cytotoxic anticancer agents.

The use of stimuli-responsive hydrogels in the treatment of oral cancer is still not supported by defined rules, regulations, and standards, despite the modern pharmaceu-tical industry’s rapid advancement. The U.S. Food and Drug Administration (FDA) has only authorized some hydrogel for use as facial correction, filler, and contact lens. The rising promise of several illness therapies is already visible in hydrogel materials. Unfortunately, few of the novel remedies that have been created and published have been tested in clinical studies. In the Clinical Trials Gov database (https://clinicaltrials.gov/ (accessed on 27 October 2022)), there are 636 completed clinical trials and some clinical trials that are now recruiting participants to conduct application tests for various hydrogel products. Of them, only 59 are concerned with the treatment and prognosis of cancer. At present, the use of smart hydrogel drug-delivery methods is superficial, and in most cases it was applied to the skin and ocular surface [199,200]. The safety of the injection route used in the treatment of oral cancer needs to be fully confirmed. At the same time, many challenges, including biocompatibility, toxicology, and biodegradation, remain to be solved. The safety of the injection route used in the treatment of oral cancer needs to be fully confirmed. At the same time, many challenges, including biocompatibility, toxicology, and biodegradation, remain to be solved.

In conclusion, smart hydrogel-based drug delivery systems have a significant effect on oral cancer chemotherapy and can reduce damage to normal tissue. Future research can fully utilize the benefits of the drug delivery technology and include it in other oral cancer treatments to address shortcomings, improve curative effects, and reduce costs. These advantages provide a new strategy for the chemotherapy of oral cancer.

## Figures and Tables

**Table 1:** The differences between the various types of stimulus-responsive hydrogels.

Types	Principle of Change under Stimulus	Advantages	Examples	Ref.
Temperature-responsive	Change of hydrophobic interaction.	Biocompatibility, easy to function with drug molecules, controlled degradation.	Poloxamer, Pluronic, HA, PPZ, PLGA; PEG.	[182,183,184,185,186,187,188,189]
Light-responsive	Destruction of seepage balance.	Space–time control of drug release;	HPMC, IR820/methylcellulose hydrogels.	[143,190]
Enzyme-responsive	Can be formed or degraded under the catalysis of related enzymes.	Can realize molecular recognition, high affinity, mild stimulus.	MMP-responsive Peptide-crosslinked PEG hydrogels.	[191,192,193,194]
pH-responsive	Change of hydrophobicity; increase of electrostatic repulsion.	Biocompatibility, Suitable for acidic tumor microenvironment, strong electrostatic interaction, strong stability.	PEI, PAM, PAAm, PDMAEMA, PDEAEMA, PMAA, PVAm	[195,196,197,198]

Abbreviation: Polyorganophosphazene (PPZ); poly (lactic acid-co-glycolic acid) (PLGA); Polyethyleneglycol-diacid (PEG); Polymers hydroxypropyl methylcellulose (HPMC); Poly(ethylene imine) (PEI); Poly(acrylamide) (PAAm); Poly(N,N’-dimethyl aminoethyl methacrylate) (PDMAEMA); Poly(N,N’-diethyl aminoethyl methacrylate) (PDEAEMA); Poly(2-aminoethyl methacrylate) (PMAA); Poly(vinylamine) (PVAm).
